# Whole-Genome Sequencing of Procyonids Reveals Distinct Demographic Histories in Kinkajou (*Potos flavus*) and Northern Raccoon (*Procyon lotor*)

**DOI:** 10.1093/gbe/evaa255

**Published:** 2020-12-17

**Authors:** Mirian T N Tsuchiya, Rebecca B Dikow, Klaus-Peter Koepfli, Paul B Frandsen, Larry L Rockwood, Jesús E Maldonado

**Affiliations:** 1 Data Science Lab, Office of the Chief Information Officer, Smithsonian Institution, Washington, DC, USA; 2 Center for Conservation Genomics, Smithsonian Conservation Biology Institute, National Zoological Park, Washington, DC, USA; 3 Smithsonian-Mason School of Conservation, George Mason Univeristy, Front Royal, VA, USA; 4 Smithsonian Conservation Biology Institute, Center for Species Survival, National Zoological Park, Washington, DC, USA; 5 Department of Plant & Wildlife Sciences, Brigham Young University, Provo, UT, USA; 6 Department of Biology, George Mason University, Fairfax, VA, USA

**Keywords:** mammal genomics, genome assembly, demographic history

## Abstract

Here, we present the initial comparison of the nuclear genomes of the North American raccoon (*Procyon lotor*) and the kinkajou (*Potos flavus*) based on draft assemblies. These two species encompass almost 21 Myr of evolutionary history within Procyonidae. Because assemblies greatly impact downstream results, such as gene prediction and annotation, we tested three de novo assembly strategies (implemented in ALLPATHS-LG, MaSuRCA, and Platanus), some of which are optimized for highly heterozygous genomes. We discovered significant variation in contig and scaffold N50 and L50 statistics and genome completeness depending on the de novo assembler used. We compared the performance of these three assembly algorithms in hopes that this study will aid others looking to improve the quality of existing draft genome assemblies even without additional sequence data. We also estimate the demographic histories of raccoons and kinkajous using the Pairwise Sequentially Markovian Coalescent and discuss the variation in population sizes with respect to climatic change during the Pleistocene, as well as aspects of their ecology and taxonomy. Our goal is to achieve a better understanding of the evolutionary history of procyonids and to create robust genomic resources for future studies regarding adaptive divergence and selection.

**Significance**

Procyonids are among the most well-known mammals across their range, and include coatis, olingos, raccoons, and kinkajous. Here, we present the draft genomes of the kinkajou (*Potos flavus*) and the northern raccoon (*Procyon lotor*), the first procyonid assemblies deposited to GenBank. We also evaluated how different assembly strategies affect genome contiguity and completeness. These are particularly important for researchers who do not have access to high-quality DNA samples for their study species, and for whom long-read technologies are still out of reach. These genomes are also a useful resource for future studies on the development of species-specific markers for the study of evolutionary history, population genomics, adaptive divergence, and disease ecology of procyonids and can add to the comparative power of larger-scale mammal genomics studies.

## Introduction

**Fig. 1:**
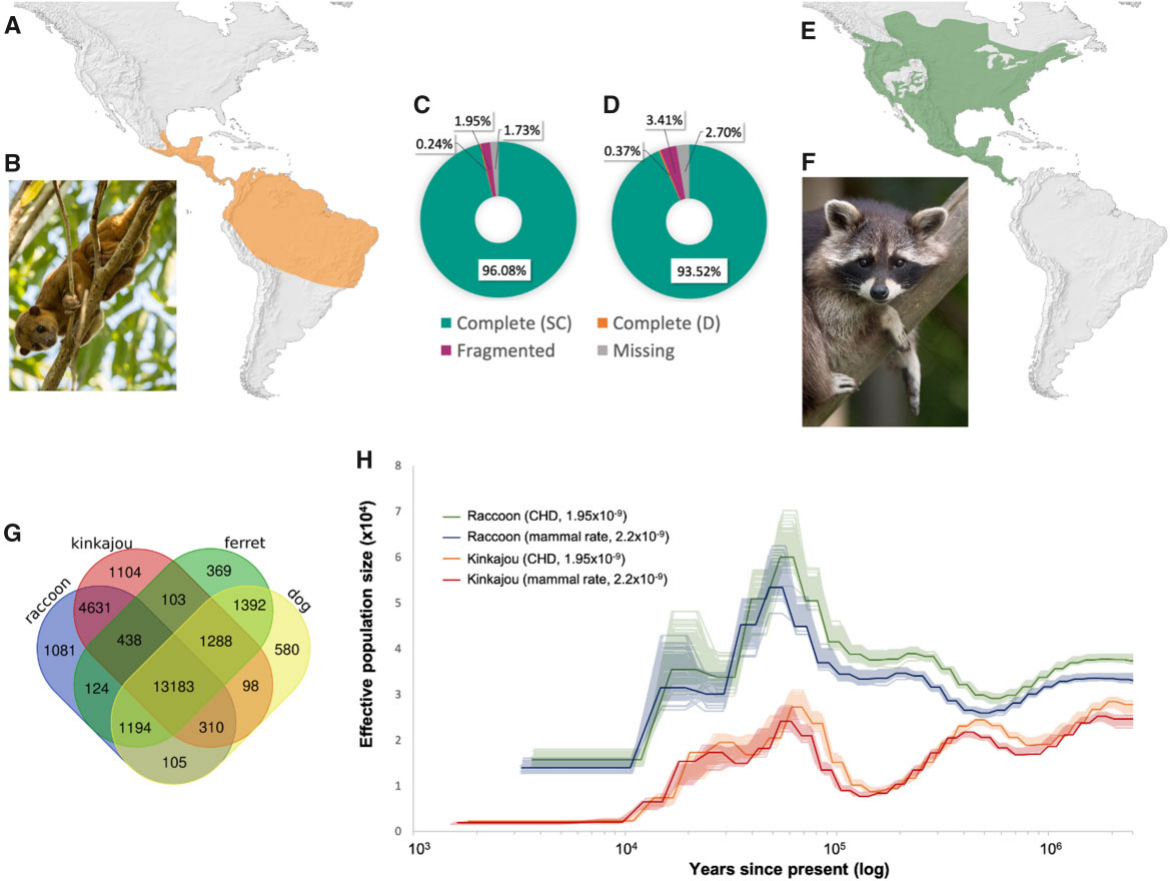
(*A*) Kinkajou range distribution. (*B*) Image of a kinkajou, *Potos flavu*s (Photo by Wim Hoek ©). (*C*) BUSCO results for the kinkajou genome. (*D*) BUSCO results for the raccoon genome. BUSCO legend: SC, single copy; D, duplicate. (*E*) Northern raccoon range distribution. (*F*) Image of a northern raccoon, *Procyon lotor* (Photo by Bernard Landgraf, Wikimedia Creative Commons). (*G*) Venn diagram depicting a comparison of orthogroups identified in each species by Orthofinder. (*H*) Effective population size reconstruction estimated with PSMC for both species, using two different mutation rates (see legend).

The Procyonidae (Gray 1825) is one of the four families of the superfamily Musteloidea within the mammalian order Carnivora. It comprises six genera of medium-sized mammals distributed in the New World (Wozencraft et al. 2005), and their representatives include raccoons, coatis, olingos, ringtails, and kinkajous. Kinkajous (*Potos flavus*) are distributed in forested habitats from southern Mexico to central South America and are highly frugivorous in their diet (Kays 1999; Kissling et al. 2014; Pineda-Munoz and Alroy 2014). In contrast, raccoons are distributed from southern Canada to Costa Rica, are well adapted to cold weather, and are omnivorous (fig. 1; Lotze and Anderson 1979; Ford and Hoffmann 1988; Prange and Prange 2009). Procyonids harbor extensive ecological, morphological, and physiological diversity and provide an ideal model system for comparative genomic analyses in revealing the genomic landscape of adaptive divergence (Koepfli et al. 2007).

Compared with other families of the mammalian order Carnivora for which genome assemblies have been generated during the last 15 years, assemblies of procyonid species have been produced only very recently (e.g., https://www.dnazoo.org), but with relatively low contig N50s. In this study, we present the draft genome assemblies for the kinkajou and the northern raccoon. Despite the decreasing costs in long-read sequencing, those might still be cost-prohibitive to many biodiversity researchers. Thus, we evaluated different assembly methods regarding their completeness, contiguity, and synteny to one another, intending to provide alternatives to improving genome assemblies without adding more data. We also compare the estimated demographic histories of the raccoon and kinkajou and discuss the findings in light of their taxonomy and ecological characteristics.

## Materials and Methods

### Sampling and DNA Sequencing

We obtained a tissue sample from a voucher male specimen of a northern raccoon deposited in the National Museum of Natural History Biorepository (*Procyon lotor*, USNM 570161, Partlow VA, USA). Heart tissue was obtained from a kinkajou deposited in the Frozen Zoo at the San Diego Zoo’s Institute for Conservation Research (*P. flavus*, unspecified location, Request number: BR2015034). We extracted genomic DNA from three raccoon tissue replicates using a DNeasy Blood and Tissue kit (Qiagen, Valencia, CA, USA). Genomic DNA was then extracted from the kinkajou tissue using the standard phenol–chloroform method (Sambrook et al. 1989) and precipitated with 96% ethanol, and then resuspended in 1× TE pH 8.0 buffer. This extraction was performed at the San Diego Zoo’s Institute for Conservation Research. Sample quality evaluation, library preparation, and whole-genome sequencing were performed by Psomagen, Inc. (Rockville, MD, USA). Samples were checked for quality (Quant-iT PicoGreen dsDNA Assay Kit using Victor 3 fluorometry), purity (ratio 260/280 nm on Nanodrop), and DNA condition (1% agarose gel). For each sample, three paired-end libraries with a 350 bp insert size (TruSeq DNA PCR-Free Library Prep Kit, Illumina, USA) and two mate-pair libraries with fragment sizes of 3 kb and 8 kb (Nextera Mate Pair Library Prep Kit, Illumina, USA) were prepared. Each library was paired-end 2× 100 bp sequenced on a Illumina Hiseq 2000 lane (Illumina, San Diego, CA, USA).

### Genome Assembly, Heterozygosity, and Size

We used TrimGalore (Krueger 2015) to remove adapter sequences and filter out reads with Phred quality scores below 20. The filtered reads were used to estimate heterozygosity, genome size, and duplication content using Genomescope (Vurture et al. 2017), based on the K-mer histograms generated using Jellyfish (Marçais and Kingsford 2011). We used MitoFinder (Allio et al. 2020) to extract and assemble the mitochondrial genomes of both species, using published *P. lotor* mitochondrial genome as a reference (GenBank Accession Number: AB462049).

For the nuclear genome, we evaluated the performance of three different de novo assembly algorithms: ALLPATHS-LG (Butler et al. 2008; Maccallum et al. 2009; Gnerre et al. 2011), MaSuRCA (Zimin et al. 2013), and Platanus (Kajitani et al. 2014), with default parameters. All scaffolds <500 bp were removed. The program assembly_stats 0.1.4 (Trizna 2020) was used to generate summary statistics for each assembly (supplementary tables S1 and S2, Supplementary Material online). We used Kraken v.2.0 (Wood and Salzberg 2014) to assess the presence of contamination and removed the scaffolds that were classified as bacteria. We assessed genome completeness using the software BUSCO v3 (Simão et al. 2015; Waterhouse et al. 2018), with the mammalian data set (mammalia_odb9, 4104 BUSCOs) and compared the different assemblies to each other and to other Carnivoran genomes available on GenBank.

Whole-genome alignments of the kinkajou and northern raccoon assemblies were constructed using Cactus (Armstrong et al. 2020). Each alignment included all three genome assemblies as well as the chromosome-length Hi-C assemblies of both species generated by DNA Zoo (Dudchenko et al. 2017, 2018). We then used Ragout (Kolmogorov et al. 2014, 2018) to assign scaffold assemblies to pseudochromosomes using the Hi-C genomes as references. Pseudochromosome scaffolds were then aligned to the Hi-C assemblies as well as the domestic dog reference genome (BioSample: SAMN02953603) using nucmer (Delcher et al. 2002) in order to assess synteny. Nucmer alignments were visualized as dot plots using the DNAnexus Dot tool (https://dnanexus.github.io/dot/).

### Genome Annotation and Variant Calling

Genome annotation and variant calling workflows were described in detail in Tsuchiya et al. (2020). Briefly, we identified, annotated, and masked repetitive and low complexity DNA sequences using RepeatMasker 4.0.6 (Smit et al. 2013), with the database of Carnivora repeats (RepeatMasker Combined Database Dfam 3.0). We used AUGUSTUS (Stanke et al. 2006) to make ab initio and evidence-based predictions using gene models trained on each species during the BUSCO analyses. We annotated both strands of the masked assembly using protein hints from five Carnivora species: domestic dog (*Canis lupus familiaris*, PRJNA13179), sea otter (*Enhydra lutris*, PRJNA407952), stoat (*Mustela erminea*, PRJNA602914), domestic ferret (*Mustela putorius furo*, PRJNA59869) and North American river otter (*Lontra canadensis*, PRJNA611578). AUGUSTUS hints were generated using BLAT (Kent 2002), with higher priority given to the domestic dog hints, since the domestic dog assembly is more completely annotated compared with the other available species. The amino acid sequences of the AUGUSTUS gene models were queried against the nonredundant protein database using blastp (nr, *e*-value 1*e*-4, max_target_seqs = 10). The blastp results were used as input for Blast2GO v.5.2.5 (Götz et al. 2008), with the final gene set for each species corresponding to the gene models with functional annotations identified by the Blast2GO suite. We used the amino acid sequences from the Blast2GO annotated genes to identify orthologous groups using Orthofinder 2.4 (Emms and Kelly 2019) and compared those to the domestic ferret and the domestic dog protein sequences listed above.

To call variants, we first mapped filtered reads to the assemblies using Bowtie2 (Langmead and Salzberg 2012) and then Samtools v1.9 (Li et al. 2009) for bam manipulation and sorting. We marked PCR duplicates using picard-tools v.2.2 (Broad Institute) and used the Haplotypecaller algorithm implemented in GATK V.3.8.1 (McKenna et al. 2010; Poplin et al. 2017) to identify heterozygous sites and generate a VCF file. We used the command bcftools stats (version 1.9 + htslib-1.9, https://samtools.github.io/bcftools/bcftools.html) to estimate the number of single nucleotide polymorphisms (SNPs) and insertions and deletions (indels).

### Ancestral Demographic Reconstructions

We used the Pairwise Sequentially Markovian Coalescent (PSMC 0.6.5, Li and Durbin 2011) to infer effective population size history for each species. The commands bcftools mpileup, bcftools call, and vcfutils vcf2fastq were used to obtain the consensus sequences for each species (Li 2011). We used PSMC default parameters for the atomic time intervals (-N25 -t15 -r5 -p “4 + 25*2 + 4 + 6”) and assessed the results with 100 bootstraps replicates. For both species, we employed the mammal average neutral substitution rates, 2.2 × 10^–09^ substitutions/site/year (Kumar and Subramanian 2002) as well as the estimated rate between cattle, human, and dog genomes, 1.95 × 10^–09^ substitutions/site/year (Liu et al. 2006), and we present the comparison between those two rates for both species. Generation time was estimated at ∼7 years for the raccoon and 11 years for the kinkajou, following (Pacifici et al. 2013).

## Results and Discussion

### Genome Assembly, Heterozygosity, and Size

We sequenced a total of 216 Gbp for the raccoon (34× coverage), and 238 Gbp for the kinkajou (48× coverage). Genomescope results show that the estimated heterozygosity for the raccoon is slightly higher than for the kinkajou (0.44% vs. 0.35%). The kinkajou genome (JAABKN000000000) was assembled into 15,701 scaffolds, totaling 2.20 Gbp, GC content of 41.58% and an N50 of 3.5 Mb. Comparatively, the raccoon genome (JAABKC000000000) was assembled in 49,250 scaffolds, totaling 2.50 Gbp, with a GC content of 41.67% and an N50 of 1.45 Mb (fig. 1, supplementary tables S1 and S2, Supplementary Material online). Independent from the assembly method used, the raccoon assemblies were more fragmented than the kinkajou ones, which could be due to its higher heterozygosity (see Asalone et al. 2020).

Pseudochromosome assemblies built in Ragout based on the progressive Cactus alignments, AGP files indicating chromosome assignments, nucmer alignments and dot plots comparing raccoon and kinkajou pseudochromosome assemblies with the domestic dog genome are available on FigShare (see Data Availability). The domestic dog and procyonids is diverged almost 50 Ma (Eizirik et al. 2010), thus it's not surprising to see so many chromosome rearrangements when we compare our assemblies to the dog genome (supplementary figs. S1 and S2, Supplementary Material online). Conversely, we observe a higher level of synteny between kinkajou and raccoon (supplementary fig. S3, Supplementary Material online). Additional procyonid genomes would permit a deeper understanding of chromosomal evolution in this family.

Among the three assembly methods tested, the Platanus assembly was the most contiguous and had the highest BUSCO scores, with the lowest number of missing genes for both genomes. Therefore, all subsequent analyses were performed with the Platanus assemblies (supplementary table S3, Supplementary Material online). BUSCO results show that both genome assemblies presented in this study have a high level of gene completeness, with only 71 of missing BUSCOs for the kinkajou and 111 for the raccoon (fig. 1, supplementary table S3, Supplementary Material online). When we compare the kinkajou and raccoon assemblies to published assemblies from eight Carnivora species (supplementary table S3 and fig. S4, Supplementary Material online), we observe that the Platanus assemblies for both species provide a significant improvement regarding genome completeness compared with the MaSuRCA and ALLPATHS-LG assemblies. In the case of the kinkajou, even though its average coverage (48×) is much lower than that of several species listed (e.g., red panda: 115×), the Platanus assembly was able to recover almost the entire BUSCO data set (supplementary table S3 and fig. S4, Supplementary Material online).

Mitofinder recovered the full mitochondrial genomes for both species. The kinkajou mitochondrial genome is 16,434 bp long, whereas the raccoon is 16,557 bp. The difference in size is mainly due to the *D-loop* region: the raccoon *D-loop* is 1,096 bp long whereas that of the kinkajou is 995 bp.

### Genome Annotation and Variant Calling

RepeatMasker estimated the repeat content at 30.25% for the kinkajou and 26.54% for the raccoon (supplementary table S4, Supplementary Material online). AUGUSTUS identified a similar number of gene models and coding DNA sequences (CDS) for both species: 67,115 gene models and 285,235 CDS for the kinkajou, and 66,962 gene models and 279,226 CDS for the raccoon. The blastp and Blast2GO results are also very similar between the two species: blastp found matches for 53% of the AUGUSTUS gene models for both species (35,571 for the kinkajou and 35,715 for the raccoon). The final GFF files include the AUGUSTUS gene models with BLAST hits and Blast2GO functional annotation, corresponding to 45% of the predicted gene models for the raccoon (29,801) and the kinkajou (29,879). In the comparative analysis performed with Orthofinder, we found a total of 178,133 genes for the domestic dog, domestic ferret, kinkajou, and raccoon, and 91.9% of those were assigned to 26,000 orthogroups (fig. 1*G*). A total of 13,183 orthogroups were shared among all species. The raccoon and kinkajou shared 4,631 exclusive orthogroups, and we identified 1,081 unique orthogroups found only in the raccoon and 1,104 in the kinkajou. Our variant calling workflow identified a higher number of SNPs for the raccoon compared with the kinkajou. We found 2,740,429 SNPs for the raccoon, and 4,528,704 for the kinkajou.

### Ancestral Demographic Reconstructions

The population size history estimates are presented in figure 1*H*. The PSMC estimates show an increase in population sizes for both species starting around 100 ka, following the Eemian interglacial period (150–115 ka), after which population sizes decline for both species. Despite the similarity in curve shapes, PSMC indicates a larger effective population size for the raccoon than for the kinkajou, with no significant differences between the two different mutation rates tested. We can infer that the differences in population size estimates are due to the fact that the northern raccoon is a single, widespread species, whereas the kinkajou is possibly multiple species currently classified as a single species. Previous studies have found little evidence of genetic structure, with extensive gene flow among raccoon populations (Cullingham et al. 2008; Santonastaso et al. 2012). On the other hand, recent evidence suggests that the genus *Potos* corresponds to potentially five to seven species instead of one (Nascimento et al. 2016). Thus, the PSMC plot may reflect the demographic history of one of those unrecognized species, with a much smaller distribution than currently assumed, and hence, a smaller effective population size.

These genomes contribute to the knowledge of the order Carnivora, a group that is relatively well-studied with the exception of certain families, such as the Procyonidae. We also showed that different assembly methods produce assemblies with very different levels of N50 contiguity and BUSCO completeness. This information is important particularly for biodiversity researchers who might not have the resources to generate long-read and very high-coverage genomes. The assemblies and annotation presented in this study offer an important resource for the development of species-specific markers for the study of evolutionary history, population genomics, adaptive divergence, and disease ecology. The raccoon genome, in particular, can be used to understand susceptibility and disease ecology of rabies and inform future management efforts related to this disease.

## Supplementary Material

Supplementary data are available at *Genome Biology and Evolution* online.

## Supplementary Material

evaa255_Supplementary_Data
