# Supplementary material for: Whole-Genome Sequencing of Procyonids Reveals Distinct Demographic Histories in Kinkajou (Potos flavus) and Northern Raccoon (Procyon lotor)
Source: Genome Biol Evol. 2020 Dec 17;13(1):evaa255. doi: 10.1093/gbe/evaa255 (PMC7851585; doi:10.1093/gbe/evaa255)
Supplement: evaa255_Supplementary_Data [file evaa255_supplementary_data.docx]

**SUPPLEMENTARY MATERIALS**

**Table S1.** Summary statistics for the kinkajou assemblies using ALLPATHS-LG, MaSuRCA and Platanus

|  | Platanus | MaSuRCA | ALLPATHS-LG |
| --- | --- | --- | --- |
| Total base pairs (Gb) | 2.20 | 2.30 | 2.04 |
| Total number | 15,701 | 66,373 | 23,301 |
| GC content | 41.58% | 41.69% | 41.52% |
| L50 | 174 | 5,676 | 1,885 |
| N50 (bp) | 3,555,714 | 117,919 | 292,086 |
| Mean (bp) | 140,639 | 34,638 | 87,776 |
| Median (bp) | 962 | 6,283 | 20,222 |
| Shortest (bp) | 500 | 500 | 878 |
| Longest (bp) | 15,435,475 | 1,006,414 | 3,906,870 |

**Table S2.** Summary statistics for the raccoon assemblies using ALLPATHS-LG, MaSuRCA and Platanus

|  | Platanus | MaSuRCA | ALLPATHS-LG |
| --- | --- | --- | --- |
| Total base pairs (Gb) | 2.25 | 2.78 | 1.79 |
| Total number | 49,250 | 273,112 | 42,326 |
| GC content | 41.67% | 41.69% | 41.46% |
| L50 | 415 | 1,995 | 4,316 |
| N50 (bp) | 1,449,398 | 383,391 | 111,530 |
| Mean (bp) | 45,711 | 10,182 | 42,330 |
| Median (bp) | 1,043 | 850 | 16,284 |
| Shortest (bp) | 500 | 500 | 881 |
| Longest (bp) | 10,598,971 | 3,432,958 | 1,833,237 |

Table S3: BUSCO scores for the kinkajou and raccoon assemblies as well as eight additional Carnivora assemblies from GenBank.

| **Figure ID** | **Family** | **Scientific Name** | **BioProject Number** | **BioSample Number** | **Assembler** | **Cov** | **Assembly Stage** | **C** | **D** | **F** | **M** | **Reference** |
| --- | --- | --- | --- | --- | --- | --- | --- | --- | --- | --- | --- | --- |
| Red Panda | Ailuridae | *Ailurus fulgens styani* | PRJNA298932 | SAMN04169031 | SOAPdenovo | 115.5x | Scaff | 3868 | 36 | 108 | 92 | Hu Y, et al. 2017 |
| African Wild Dog | Canidae | *Lycaon pictus* | PRJNA304992 | SAMN04312208 | BWA | 5.7x | Chrom | 3740 | 25 | 185 | 154 | Campana et al. 2016 |
| Dog | Canidae | *Canis lupus familiaris* | PRJNA13179 | SAMN02953603 | Arachne | 7x (S) | Chrom | 3859 | 52 | 98 | 95 | Lindblad-Toh et al. 2005 |
| Ferret | Mustelidae | *Mustela putorius furo* | PRJNA59869 | SAMN00149631 | ALLPATHS-LG | 162x | Scaff | 3885 | 26 | 103 | 90 | Peng et al. 2014 |
| Sea Otter | Mustelidae | *Enhydra lutris* | PRJNA407952 | SAMN07211055 | ABySS/Supernova | 110x | Scaff | 3903 | 43 | 77 | 81 | Jones et al. 2017 |
| Pacific Walrus | Odobenidae | *Odobenus rosmarus* | PRJNA189954 | SAMN01180787 | ALLPATHS; Atlas-link; Atlas-gapfill | 200x | Scaff | 3842 | 61 | 105 | 96 | Foote et al. 2015 |
| Raccoon (AP) | Procyonidae | *Procyon lotor* | n/a | n/a | ALLPATHS-LG | 34x | Scaff | 2191 | 11 | 859 | 1043 | This study |
| Kinkajou (AP) | Procyonidae | *Potos flavus* | n/a | n/a | ALLPATHS-LG | 48x | Scaff | 3271 | 12 | 461 | 360 | This study |
| Raccoon (M) | Procyonidae | *Procyon lotor* | n/a | n/a | MaSuRCA | 34x | Scaff | 3579 | 47 | 289 | 189 | This study |
| Kinkajou (M) | Procyonidae | *Potos flavus* | n/a | n/a | MaSuRCA | 48x | Scaff | 3450 | 23 | 444 | 187 | This study |
| Raccoon (P) | Procyonidae | *Procyon lotor* | PRJNA600180 | SAMN08536241 | Platanus | 34x | Scaff | 3838 | 15 | 140 | 111 | This study |
| Kinkajou (P) | Procyonidae | *Potos flavus* | PRJNA600180 | SAMN13795725 | Platanus | 48x | Scaff | 3943 | 10 | 80 | 71 | This study |
| Giant Panda | Ursidae | *Ailuropoda melanoleuca* | PRJNA299083 | SAMN04193337 | SOAPdenovo | 70x | Scaff | 3894 | 14 | 105 | 91 | Li et al. 2010 |
| Polar Bear | Ursidae | *Ursus maritimus* | PRJNA210951 | SAMN02729226 | SOAPdenovo | 101x | Scaff | 3872 | 18 | 125 | 89 | Liu et al. 2014 |

AP: ALLPATHS-LG

M: MaSuRCA

P: Platanus

Cov: coverage

C: Single-Copy Complete BUSCO

D: Duplicate Complete BUSCO

F: Fragmented BUSCO

M: Missing BUSCO

**Table S4:** RepeatMasker results for kinkajou and raccoon, showing the percentage of each kind of identified repetitive or low-complexity elements.

|  | Kinkajou | Raccoon |
| --- | --- | --- |
| SINEs: | 3.06% | 2.76% |
| Alu/B1 | 0.00% | 0.00% |
| MIRs | 3.02% | 2.73% |
| LINEs: | 16.69% | 14.31% |
| LINE1 | 12.35% | 10.51% |
| LINE2 | 3.78% | 3.32% |
| L3/CR1 | 0.40% | 0.36% |
| RTE | 0.14% | 0.12% |
| LTR elements: | 4.72% | 4.30% |
| ERVL | 1.71% | 1.57% |
| ERVL-MaLRs | 2.24% | 2.05% |
| ERV_classI | 0.52% | 0.46% |
| ERV_classII | 0.00% | 0.00% |
| DNA elements: | 3.04% | 2.73% |
| hAT-Charlie | 1.63% | 1.47% |
| TcMar-Tigger | 0.63% | 0.56% |
| Unclassified: | 0.03% | 0.02% |
| Total interspersed repeats: | 27.53% | 24.13% |
| Small RNA: | 0.30% | 0.24% |
| Satellites: | 0.00% | 0.00% |
| Simple repeats: | 1.75% | 1.61% |
| Low complexity: | 0.66% | 0.55% |

**
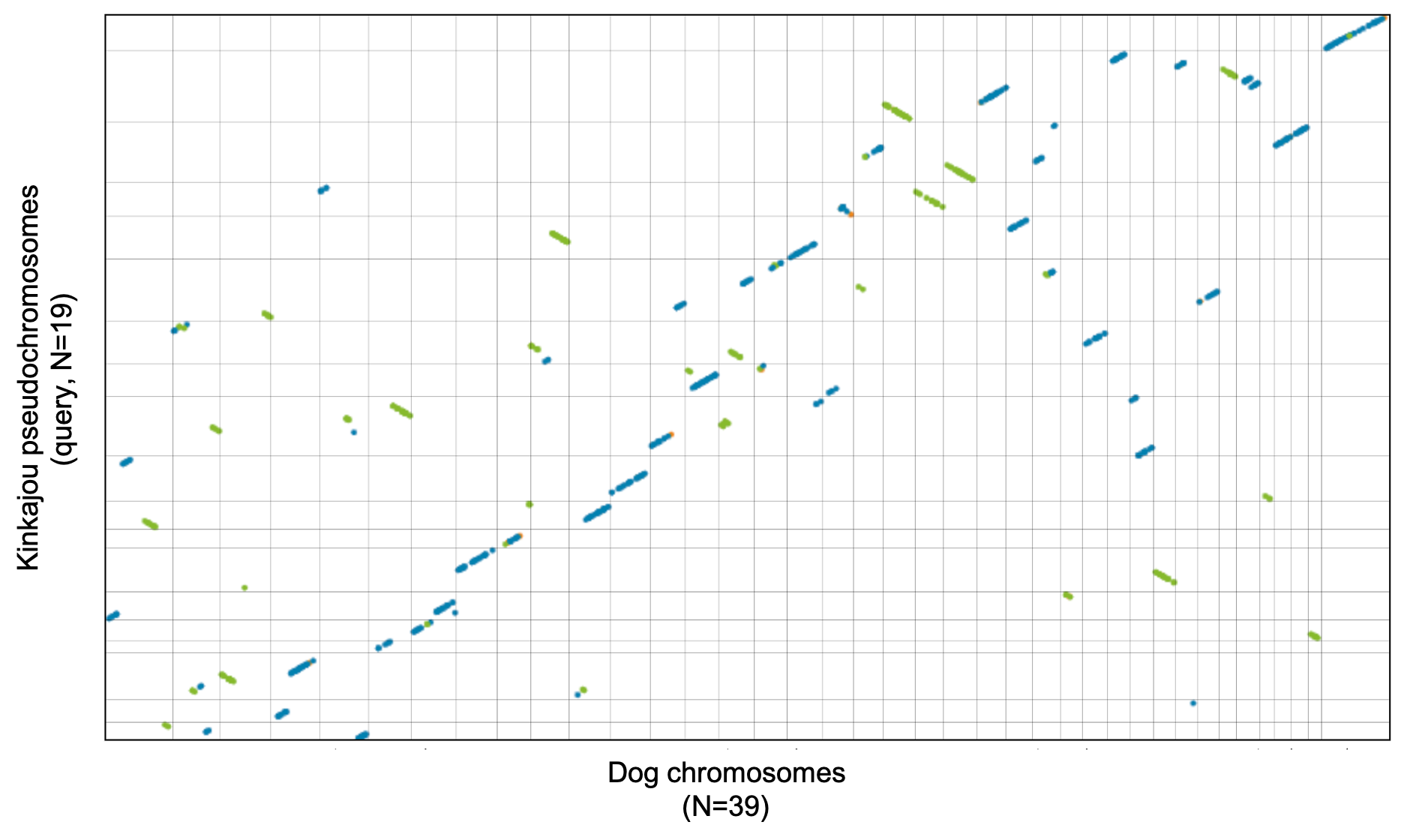
**

**Figure S1:** Dot plot of the percent identity between the dog chromosomes (x-axis) and the kinkajou pseudochromosomes (y-axis) generated using nucmer. Blue dots represent forward matches and green dots represent reverse matches.


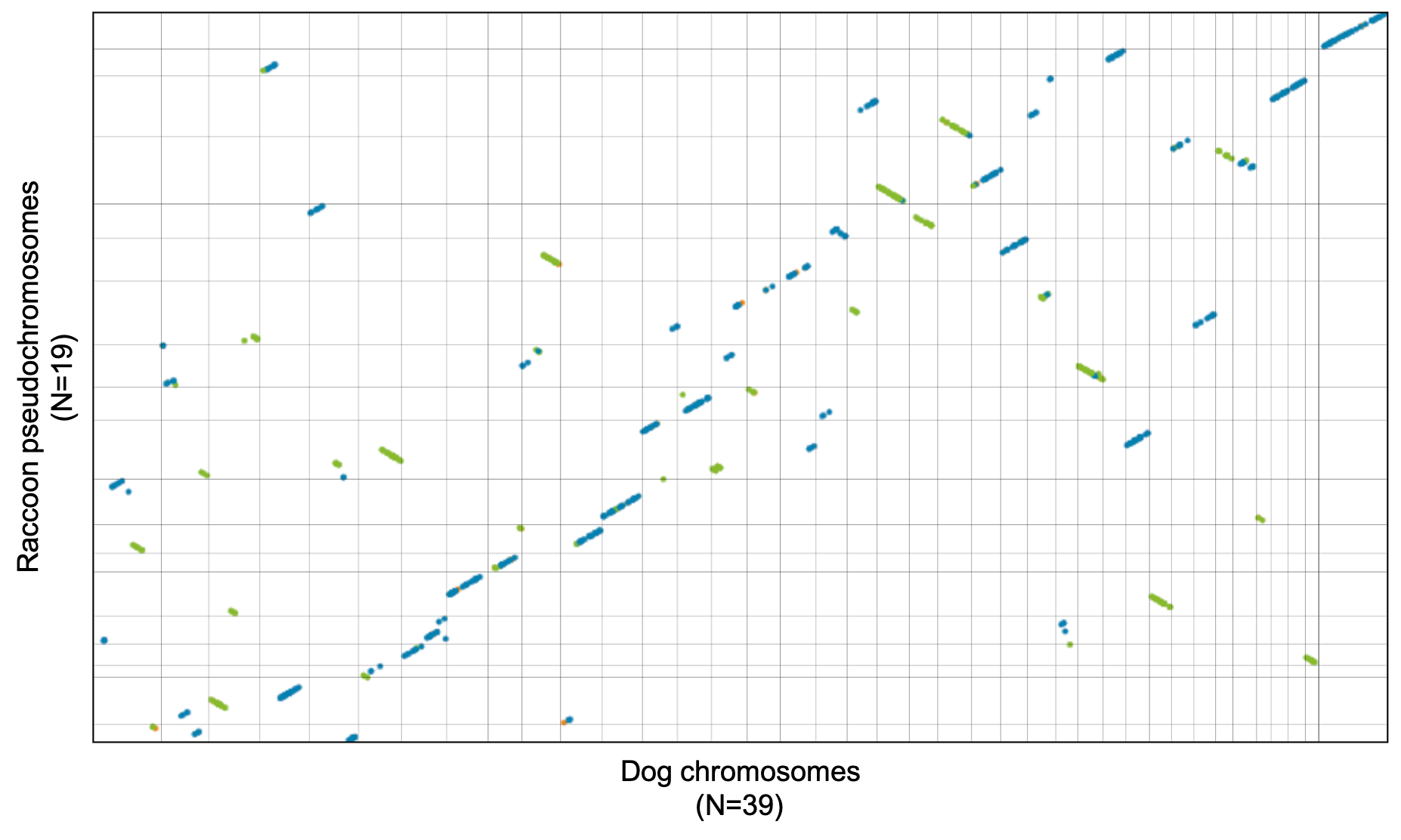


**Figure S2:** Dot plot of the percent identity between the dog chromosomes (x-axis) and the raccoon pseudochromosomes (y-axis) generated using nucmer. Blue dots represent forward matches and green dots represent reverse matches.

**
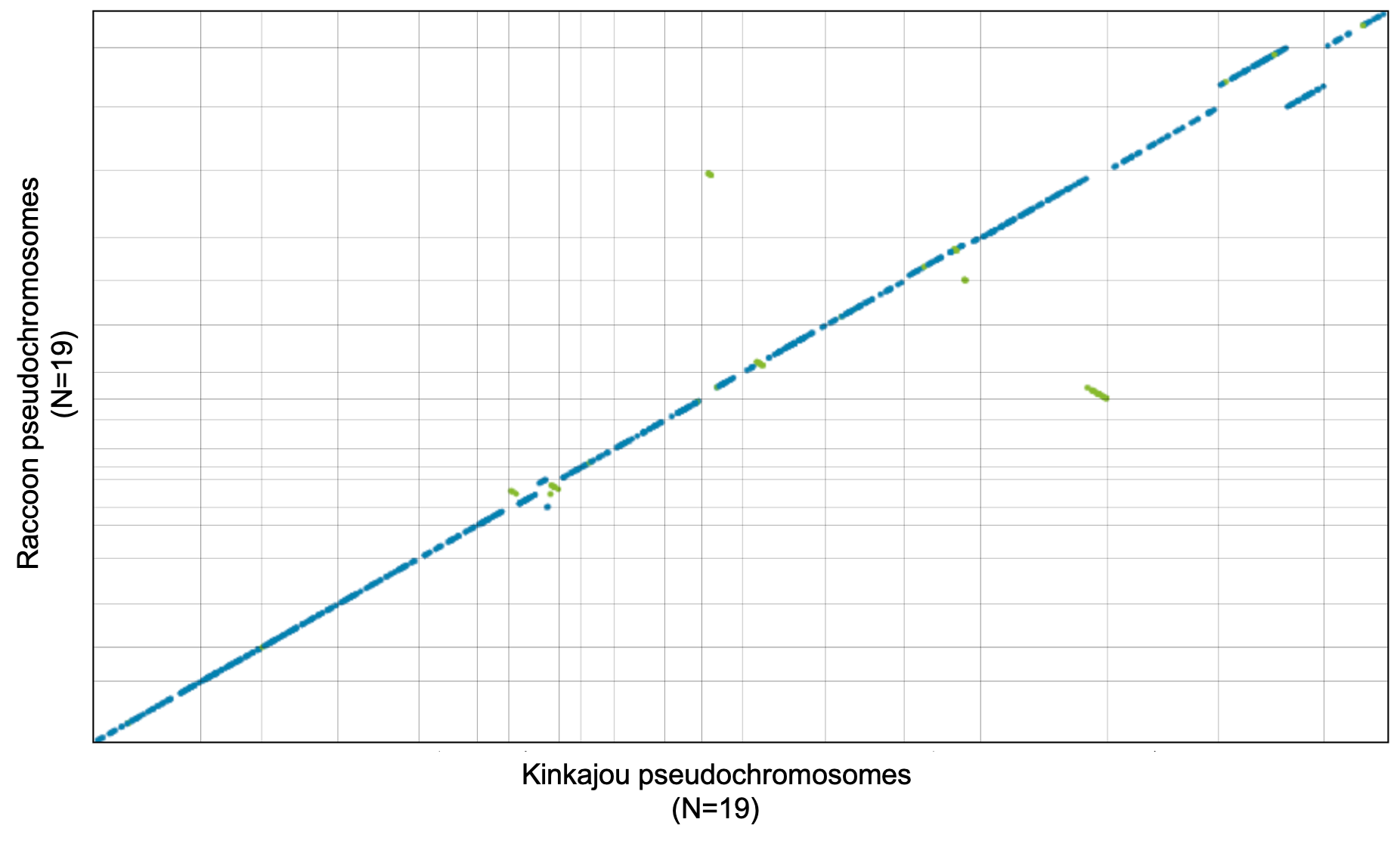
**

**Figure S3:** Dot plot of the percent identity between the kinkajou (x-axis) and the raccoon pseudochromosomes (y-axis) generated using nucmer. Blue dots represent forward matches and green dots represent reverse matches.


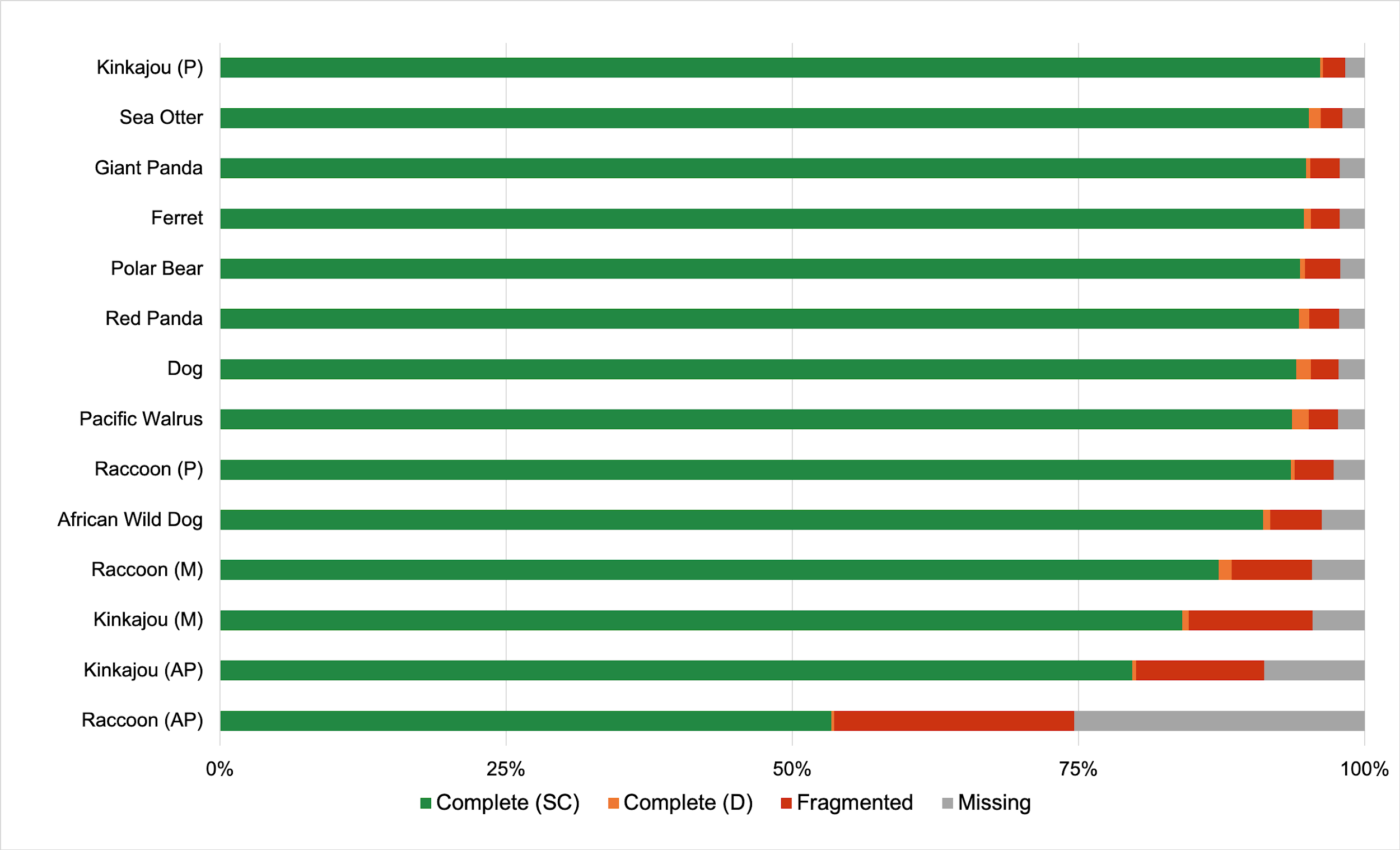


**Figure S4:** BUSCO results for Carnivora species. The number of complete BUSCOs increases from bottom to top. See Table S3 for more information about each entry. BUSCO abbreviations: SC: single copy; D: duplicate.
